# Blindness Caused by Deficiency in AE3 Chloride/Bicarbonate Exchanger

**DOI:** 10.1371/journal.pone.0000839

**Published:** 2007-09-05

**Authors:** Bernardo V. Alvarez, Gregory S. Gilmour, Silvina C. Mema, Brent T. Martin, Gary E. Shull, Joseph R. Casey, Yves Sauvé

**Affiliations:** 1 Membrane Protein Research Group, Department of Biochemistry, University of Alberta, Edmonton, Canada; 2 Department of Physiology, University of Alberta, Edmonton, Canada; 3 Department of Ophthalmology, University of Alberta, Edmonton, Canada; 4 Departments of Molecular Genetics, Biochemistry and Microbiology, University of Cincinnati College of Medicine, Cincinnati, Ohio, United States of America; University of Reading, United Kingdom

## Abstract

**Background:**

Vision is initiated by phototransduction in the outer retina by photoreceptors, whose high metabolic rate generates large CO_2_ loads. Inner retina cells then process the visual signal and CO_2_. The anion exchanger 3 gene (AE3/*Slc4a3*) encodes full-length AE3 (AE3fl) and cardiac AE3 (AE3c) isoforms, catalyzing plasma membrane Cl^−^/HCO_3_^−^ exchange in Müller (AE3fl) and horizontal (AE3c) cells. AE3 thus maintains acid-balance by removing photoreceptor-generated CO_2_ waste.

**Methodology/Principal Findings:**

We report that Slc4a3^−/−^ null mice have inner retina defects (electroretinogram b-wave reduction, optic nerve and retinal vessel anomalies). These pathologic features are common to most human vitreoretinal degenerations. Immunobloting analysis revealed that Na^+^/HCO_3_^−^ co-transporter (NBC1), and carbonic anhydrase II and CAXIV, protein expression were elevated in *Slc4a3* ^−/−^ mouse retinas, suggesting compensation for loss of AE3. TUNEL staining showed increased numbers of apoptotic nuclei from 4–6 months of age, in *Slc4a3* ^−/−^ mice, indicating late onset photoreceptor death.

**Conclusions/Significance:**

Identification of Slc4a3 as underlying a previously unrecognized cause of blindness suggests this gene as a new candidate for a subset of hereditary vitreoretinal retinal degeneration.

## Introduction

The human retina has extremely high rates of aerobic metabolism and oxygen consumption, which generates large CO_2_ loads [1]. For optical reasons, the outer retina, where photoreceptors cells are localized, is avascular [2]. Efficient elimination of the considerable CO_2_ and H^+^ load produced by photoreceptor activity is necessary to maintain intracellular (pH_i_) and extracellular (pH_o_) pH homeostasis. Aspects of the phototransduction cascade, including photoreceptor light-sensitive currents [3], cGMP content of photoreceptors [4], and ion channels activity [5], are highly sensitive to pH.

Transient changes of intracellular pH (pH_i_) and extracellular pH (pH_o_) upon synaptic activity in the retina influence the response properties of neuronal circuits. Activity of plasma membrane acid and base transporters in neurons and glial cells of the retina regulate those changes. Na^+^-independent Cl^−^/HCO_3_^−^ exchangers (AE) likely contribute to maintain ionic homeostasis in the retina [6].

The AE family comprises ten genes [7] [8]. One of them, the AE3 *gene (SLC4A3)*, encodes the full length AE3 (AE3fl) and the cardiac AE3 (AE3c) isoforms generated by alternative promoter usage [9]. The predicted cardiac AE3 (AE3c) polypeptide is 1030 amino acids in length and approximately 120 kDa, while the AE3fl variant consists of 1227 amino acids and ∼160 kDa. The C-terminal 957 amino acids of both polypeptides are identical, but AE3c contains a unique N-terminal sequence of 73 amino acids, which replaces the first 270 amino acids of AE3fl. AE3 catalyzes electroneutral Cl^−^/HCO_3_^−^ exchange across the plasma cell membrane, regulating [Cl^−^]_i_, [HCO_3_^−^]_i_, pH_i_, and volume [8]. In the central nervous system, neuronal and glial brain cells express only AE3fl isoform. Conversely, the inner retina, which is also part of the central nervous system, expresses both AE3fl and AE3c isoforms, in neuronal and glial cells [10].

To explore the role of proton trafficking in the central nervous system, it is important to complement physiological studies with its molecular counterpart. Therefore, we studied the functional role of AE3 in the inner retina, using a mouse model with a target disruption of the *Slc4a3* gene.

While inheritance patterns have been clearly demonstrated for hereditary vitreoretinal degenerations (HVD), most genes have yet to be identified. In the few cases for which genes have been discovered, there is a very clear correlation between the function of the gene and the associated phenotype. The present study of the AE3 knockout mouse provides further evidence that AE3 plays an essential role in catalyzing Cl^−^/HCO_3_^−^ exchange across the plasma membrane of Müller and horizontal cells of the inner retina [10]. Therefore, AE3 contributes to the removal of photoreceptor-generated CO_2_ waste, which contributes to maintaining acid-balance in the inner retina.

We report that mice with homozygous disruption of *Slc4a3* present with inner retinal defects and late onset photoreceptor death, which are the pathologic features of most human HVD [11].

## Methods

### Animals

The animals were housed and handled with the authorization and supervision of the Institutional Animal Care and Use Committee at the University of Alberta. Experiments were carried out in accordance with the guidelines laid down by the NIH regarding the care and use of animals for experimental procedures (NIH Publications No. 80-23, revised 1996). The procedures also conformed to the ARVO Statement for the Use of Animals in Ophthalmic and Vision Research. All efforts were made to minimize the number of animals used and their discomfort.

### Generation and genotyping of *Slc4a3* ^−/−^ mice

The *Slc4a3* gene was disrupted by replacing a region of the gene that contained most of exon 6, intron 6, the cardiac-specific first exon and promoter, and exon 7 with the neomycin resistance gene (GE Shull, manuscript in revision). The region deleted contained codons 209–319 of the long form of AE3 and codons 1–72 of the cardiac-specific form. Expression of the cardiac-specific mRNA was eliminated. Due to the introduction of premature stop codons and frameshifts, the open reading frames of any mRNAs transcribed from the promoter for the longer variant of AE3 do not include the codons for amino acids 209–1227, which contain the anion transport domains. Genomic DNA was extracted from 3.0 mm ear notch biopsies with a Qiagen Kit (Qiagen Inc., ON, CA) and this DNA was used for genotyping by polymerase chain reaction. For the detection of the *wild type* allele (+) the oligonucleotides AE3wt.for 5′-GAT GAA GAT GAC AGC CCA GGC CTT CC, and AE3wt.rev 5′-CCG GCT CTT CTG TGT GGA GAT TCG GG, were used as forward and reverse primers, and amplified a 593 bp fragment. The forward primer corresponds to a portion of the deleted AE3 gene; thus the mutant allele was not amplified. The AE3wt.for primer, and AE3dNEO.rev primer, 5′-GAC AAT AGC AGG CAT GCT GG amplified a 654 bp fragment of the mutant allele (−).

### Preparation of mouse retina membranes

Freshly isolated mouse retinas were homogenized by 12 strokes of a Dounce homogenizer in 0.5 ml/retina of ice-cold 0.32 M sucrose, 1 mM EGTA, 0.1 mM EDTA, 10 mM HEPES, pH 7.5, containing protease inhibitors (MiniComplete, Roche). Homogenates were centrifuged at 1,440 g for 5 min in a Beckman G5-6K centrifuge. Supernatants were removed and centrifuged at 66,700 g for 30 min at 4°C in a Beckman TLA 100.4 rotor. Resulting membrane fraction was resuspended in 25 µl/retina, of PBS (140 mM NaCl, 3 mM KCl, 6.5 mM Na_2_HPO_4_, 1.5 mM KH_2_PO_4_, pH 7.5). Non-membranous fractions were kept for whole retinal lysate preparation. Protein was quantified by Bradford assay, and 50 µg of protein used for immunoblots.

### Protein Expression

Expression constructs for human CAII[12], mouse CAXIV[13] and rat NBC1[14], have been described previously described. CAII, CAXIV, and NBC1 proteins were expressed by transient transfection of HEK293 cells[15], using the calcium phosphate method[16]. Cells were grown at 37°C in an air/CO_2_ (19∶1) environment in high glucose Dulbecco's Modified Eagles Medium (DMEM), supplemented with 5% (v/v) fetal bovine serum and 5% (v/v) calf serum.

### Immunodetection

HEK293 cells were mock-transfected, or individually transfected with human CAII, mouse CAXIV, or rat NBC1, cDNAs. Two days post-transfection, cells were washed in PBS buffer, and lysates of the whole tissue culture cells were prepared by addition of 150 µl 2× SDS sample buffer ((4% (w/v) sodium dodecyl sulfate, 0.13 M Tris, 2% (v/v) 2-mercaptoethanol, pH 6.8) to 60 mm Petri dish. Samples (50 µg protein for HEK293 cell lysates, 50 µg protein for whole retinal lysates, and 50 µg protein for mouse retina membranes) were resolved by SDS-PAGE on 8–10% acrylamide gels [17]. Proteins were transferred to polyvinylidene fluoride (PVDF) membranes, and then incubated with rabbit anti-CAII ((H-70, Santa Cruz (SC), CA, 1∶1000 dilutions), goat anti-CAXIV (N-19, SC, CA, 1∶500), rabbit anti-AE3 (AP3, 1∶1000 dilution), rabbit anti-AE3c (1∶1000 dilution) [15], or mouse anti-α-tubulin (TU-02, SC, CA, 1∶1000 dilution), antibody. Polyclonal anti-NBC1 antibody was generated by immunizing rabbits with a peptide corresponding to the conserved C-terminal sequence of mouse NBC1 (COOH-DSKPSDRERSPTFLERHTSC-NH_2_, Synpep, USA). Antibodies were affinity purified and cross-reactivity tested using heterologous system (individually transient transfection of HEK293 cells with NBC1 and NBC3 cDNAs, respectively). NBC1 antibody was used at a dilution of 1∶500. Immunoblots were incubated with 1∶1000 dilution of donkey anti-rabbit IgG (SC, CA), or mouse anti-goat IgG, or sheep anti-mouse IgG (NA931V, Amersham Biosciences, UK), conjugated to horseradish peroxidase [18]. Blots were visualized and quantified using ECL reagent and a Kodak Image Station.

### Immunostaining of mouse retinas and analysis by confocal microscopy

For immunofluorescence experiments, mice were euthanized with pentobarbital, intravenously (200 mg/kg). Eyes were excised and immediately frozen at −80°C in Shandon Cryomatrix™ (Thermo Electron Corporation, PA). Cryostat sections (20 µm thick) of retinas were then cut onto glass slides. Following washing (2×5 min with PBS), and blocking (10% Chicken Serum in PBS, 30 min), retinas were incubated with primary antibodies in PBS buffer, containing 0.5% Triton X-100 (overnight, in a humidified chamber, 25°C), washed (3×5 min in PBS) and incubated with secondary antibody as above (1 h, in a humidified chamber, 25°C). Primary rabbit polyclonal anti-AE3 (AP3) antibody [10], rabbit anti-AE3c [15], and rabbit anti-PKC-αC-20, Santa Cruz, USA), were used at 1∶100 dilution. Primary goat polyclonal anti-CAII (C14, Santa Cruz, USA), and goat polyclonal anti-CAXIV (N-19, Santa Cruz, USA), were used at 1∶100 dilution. Mouse monoclonal anti-GFAP (SMI-22, Sternberger Monoclonals Inc.), and mouse monoclonal anti-Bassoon (VAM-PS003, Stressgen), were used at 1∶1000 and 1∶500 dilutions, respectively. Secondary chicken anti-rabbit conjugated to Alexa Fluor 488 (green), or secondary chicken anti-goat conjugated to Alexa Fluor 594 (red), were used at 1∶100 dilutions, or secondary goat anti-rabbit conjugated to Alexa Fluor 488 (green), or secondary goat anti-mouse conjugated to Alexa Fluor 594 (red), was used at 1∶1000 dilutions. In control experiments, slides were incubated only with secondary antibodies (data not shown). Slides were washed three times in PBS and mounted and viewed using confocal microscopy. Immunostained retinas on slides were mounted in Prolong Anti-fade solution containing DAPI for nuclear staining (Molecular Probes, OR, USA). Slides were imaged with a Zeiss LSM 510 laser scanning confocal microscope imaging system mounted on an Axiovert 100 M controller. Images were collected using an oil immersion 43× objective, at a resolution of 0.5–0.7 µm field depth. Filtering was used to integrate the signal collected over 4–8 frames to decrease noise (scan time of 7 s/frame). Multiple sections from different mice were examined. Some retinas were stained for GFAP and imaged as above, directly on retinal flat mounts.

### Electron microscopy

Mice were euthanized as above. Eyes were fixed by transcardiac perfusion with 1% formaldehyde and 2% glutaraldehyde 0.1 M sodium phosphate, pH 7.2, then removed and fixed additionally for 1 h with 1% osmium tetroxide in 0.1 M sodium phosphate, pH 7.2. Tissues were then dehydrated and embedded in a 1∶1 mixture of Epon 812 (Tousimis) and Araldite 502 (Electron Microscopy Sciences) epoxy resins. Sections of quadrants of the retina were cut, including the optic nerve head or at 0.5 mm peripherally, and stained with 1% toluidine blue. Sections were viewed and imaged with a Hitachi Transmission Electron Microscope H-7000.

### Apoptotic nuclear staining

After eye removal as described for immunostaining, eyes were post-fixed in 4% paraformaldehyde for 1 h and then immersed in 10% (1 h), 20% (1 h), and 30% (12–16 h) sucrose. *In situ* cell death detection kit TUNEL Label Mix (Roche) was used to detect apopotic nuclei (green). Briefly, frozen 20 µm tissue sections were immersed in 20 µg/ml of proteinase K nuclease free buffer (Roche) with 10 mM Tris/HCl, pH 7.4, for 2 min at 4°C. Sections were rinsed repeatedly with phosphate-buffered saline (PBS) and then permeabilized with 0.1% Triton X-100 in 0.1% sodium citrate for 2 min on ice. After washing twice with PBS, 50 µl of the TUNEL reaction mixture was applied to the samples. Slides were incubated in a humidified chamber atmosphere for 45 min at 37°C in the dark. At the end of the incubation, slides were washed repeatedly with PBS, and mounted under coverslips, in the presence of DAPI. For control slides, TUNEL Label only was used, omitting the TUNEL enzyme in the reaction mixture, and no staining was detected.

### Funduscopy

Under ketamine (150 mg/kg i.p.) and xylazine (10 mg/kg i.p.) anesthesia, pupils were dilated with a drop of Tropicamide (Alcon Laboratory). A digital camera was used in conjunction with a 78D lens (Volk) mounted between the camera and the mouse eye. Photographs were taken using conscious mice to avoid corneal clouding. Vibrissae were gently and partially removed with fine scissors to prevent them from obscuring the photograph.

### Fluorescein angiography

Immediately after euthanasia (as described above), mice were transcardiacally perfused with 2 ml of a fresh solution of Fluorescein Isothiocyanate Dextran 500,000-conjugate (Sigma; 15 mg/ml of distilled water, filtered through 0.8 µm filter). The eyes were removed, post-fixed for 30 minutes and then retina flat mounts dissected to be post-fixed for one hour on filter paper (to force a flat shape during fixation). Tissues were mounted on slides and visualized with a Zeiss LSM 510 confocal microscope.

### Electroretinography

After overnight dark adaptation (12–15 h), animals were prepared for bilateral ERG recordings under dim red light. Under anesthesia with a mixture of ketamine (150 mg/kg i.p.) and xylazine (10 mg/kg i.p.), the head was secured with a stereotaxic holder and the body temperature was maintained at 38°C, using a homeothermic blanket. Pupils were dilated using 1% Tropicamide. A drop of 0.9% saline was applied on each cornea to prevent dehydration and to allow electrical contact with the recording electrode (gold wire loop). A 25-gauge platinum needle inserted subdermally behind each eye, served as reference electrode. Amplification (at 1–1000 Hz bandpass, without notch filtering), stimulus presentation, and data acquisition were provided by the Espion E^2^ system from Diagnosys LLC (Lowell, MA). Stimuli consisted of single white (6500 K) flashes (10 µs duration), repeated 3–5 times to verify the responsiveness reliability. For intensity responses, stimuli were presented at 19 increasing intensities varying from −5.7 to 2.9 log cds/m^2^ in luminance. To allow for maximal rod recovery between consecutive flashes, inter-stimuli-intervals was increased (as the stimulus intensities were progressively increased) from 10 s at lowest stimulus intensity up to 2 min at highest stimulus intensity. Amplitude of the b-wave was measured from the a-wave negative peak up to the b-wave positive apex, and not up to the peak of oscillations, which can exceed the b-wave apex. Following 10 min of photopic adaptation (30 cd/m^2^ background), cone-driven intensity responses were studied, using single flashes with intensities ranging from −1.6 to 2.9 log cds/m^2^ along 11 steps of incremental intensities. Flicker ERG was then recorded, starting at 3 Hz, then 5 Hz and up to 45 Hz (along steps of 5 Hz) to establish the critical flicker fusion, according to 10 µV criterion amplitude. Peak-to-peak amplitudes were plotted as a function of the flicker frequency.

### Statistics

Data points represent mean±s.e.m. Statistical significance between groups was assessed using ANOVA, with P<0.05 considered significant.

### URL

Detailed information regarding the potential genes causing vitreoretinal degenerations can be obtained using the web-based server, OMIM (Online Mendelian Inheritance in Man). http://www.ncbi.nlm.nih.gov/entrez/query.fcgi?db = OMIM

## Results

**Figure 1:**
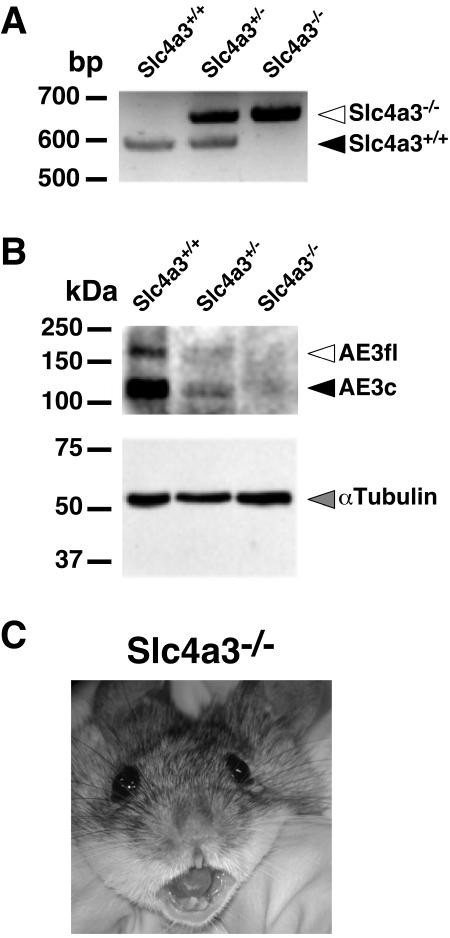
Loss of the wild-type *Slc4a3* allele and AE3 protein expression in *Slc4a3* gene-targeted mice. (A) PCR genotyping of mutant mice using genomic DNA extracted from ear notch biopsies. Open and filled arrows identify mutant and wild-type alleles, respectively. (B) Immunoblot analysis of protein samples prepared from isolated retinas of wild-type (*Slc4a3* ^+/+^), heterozygous (*Slc4a3* ^+/−^) and null (*Slc4a3* ^−/−^) mice using a C-terminal antibody to AE3 (*top panel*), which recognizes both AE3 isoforms. Open and filled arrows show full length (AE3fl), and cardiac (AE3c) AE3 isoforms, respectively. Parallel blot of protein samples from above were probed with α-tubulin as a loading marker (gray arrow) (*bottom panel*). (C) Facial characteristics of 8-month old *Slc4a3* ^−/−^ mouse.

To explore the role of AE3 in the retina we analyzed a mouse model with a targeted disruption of *Slc4a3*, in which mRNAs encoding both AE3 full-length (AE3fl) and AE3c variants had been disrupted. PCR-based genotyping distinguished *Slc4a3* wild type (+/+), heterozygous (+/−) and null (−/−) mice (Fig. 1A). Immunoblots revealed that AE3 was expressed only in *Slc4a3* ^+/+^ *Slc4a3* ^+/−^ mice (Fig. 1B). *Slc4a3* ^−/−^ mice developed normally, and were behaviorally and anatomically undistinguishable from their *Slc4a3* ^+/+^ littermates. Mutations in human *SLC4A4*, another HCO_3_^−^ transporter (NBC1, Na^+^/HCO_3_^−^ co-transporter), have been associated with short stature, poor dentition, proximal renal tubular acidosis, and bilateral cataracts with corneal opacity and late blindness onset in humans [19]–[21]. *Slc4a3* ^−/−^ mice, however, were fertile and had normal corneas and dentition with no major systemic defects (Fig. 1C).

**Figure 2:**
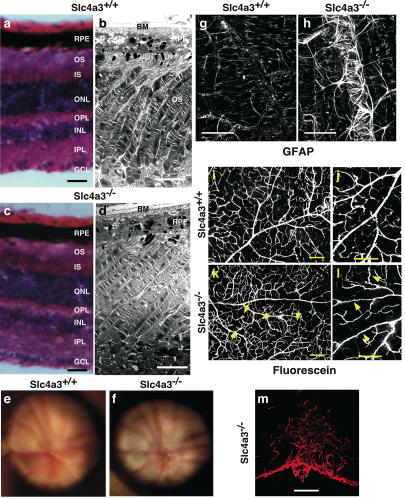
Histological analysis, funduscopy, and immunohistochemistry examination of the *Slc4a3* ^−/−^ mouse retina. Retina cross-sections (20 µm-thick) from *Slc4a3* ^+/+^ (a) and *Slc4a3* ^−/−^ (b) mice stained with hematoxylin-eosin, scale bar = 50 µm. Electron micrographs of *Slc4a3* ^+/+^ (c) and *Slc4a3* ^−/−^ (d) mouse retinas, scale bar = 5 µm; OS = outer segment; IS = inner segment; ONL = outer nuclear layer; OPL = outer plexiform layer; INL = inner nuclear layer; IPL = inner plexiform layer; GCL = ganglion cells layer. Fundus photos in 8-month-old *Slc4a3* ^+/+^ wild type (e) and *Slc4a3* ^−/−^ littermate mouse (f). Confocal microscopic pictures of retina flat mounts stained with GFAP (g, h), scale bar = 50 µm. Confocal microscopic reconstructions (*z*-stack of 50 µm depth) of fluorescein-filled blood vessels in retina flat mounts showing several loops indicative of shunting between venule and arteriole (arrows) in 8 month old *Slc4a3* ^−/−^ (k) not seen in WT (i); at higher magnification, tortuous vessels are obvious in *Slc4a3* ^−/−^ (j) compared with straight trajectories in *Slc4a3* ^+/+^(l), scale bar = 100 µm. Confocal fluorescence image of six-month old *Slc4a3* ^−/−^ mouse retina (m), stained with anti-glial fibrilar acidic protein (GFAP) antibody, at the level of the optic nerve head (anterior lamina cribosa), scale bar = 50 µm.

Consistent with anatomical observations in an independently generated *Slc4a3*-null mouse line [22], *Slc4a3* ^−/−^ retinas appeared normal with all layers present and of comparable thickness to age-matched *Slc4a3* ^+/+^ littermates at 4 months of age (Fig. 2a, 2c). While light microscopic studies revealed a normal retina, they are not optimal for assessing the integrity of outer retina component such as outer segment discs and the intricate relationship between outer segments and retinal pigment epithelial cells. Ultrastructural analysis showed the presence of full-length outer segments with invaginating retinal pigmented epithelium villi as well as normal appearance of Bruch's membrane. The discs from the outer segments were perfectly aligned (Fig. 2b, 2d). Together, the light and electron microscopy data confirm the absence of any gross retina morphology disturbances in *Slc4a3*-null mice.

The absence of any cataract formations in the eyes of *Slc4a3*-null mice allowed additional anatomical analysis using funduscopy (Fig. 2e, 2f). The retina, optic disc, choroid, and blood vessels were comparable between mutant and wild type mice. There were also no signs of any pigment migration or vitreous condensation.

To obtain a more detailed scrutiny of the retina, we probed specific structures on retinal flat mounts. Immunolabeling of astrocytes with specific glial fibrillary acidic protein (GFAP) antibody, showed that inner retina vessels were wrapped by dense astrocytic processes at 8 months of age in *Slc4a3* ^−/−^ mice (Fig. 2g, h). This pathological feature, which was absent in WT mice, is analogous to the vascular sheathing previously described on histological sections of human retinas [23]. This observation prompted us to characterize inner retina blood vessels in greater detail than possible with funduscopy. Transcardiac perfusion with fluorescein confirmed normal appearance of inner retina vessels in 8-month-old WT mice (Fig. 2i, j). Conversely, age-matched *Slc4a3* ^−/−^ mice had inner retina blood vessels that formed sporadic loops (Fig. 2k). These loops were typical of pathological cases in which venules and arterioles are shunted, i.e. not separated by capillaries. Overall, inner retina blood vessels followed tortuous trajectories (Fig. 2l) and the major vessels in the central area of the retina tended to have thinner diameters.

Detailed characterization of the optic disc, beyond the level possible with funduscopy was undertaken using GFAP immunolabeling of astrocytes on cross sections of the retina. Astrocytic processes had marked disorganization at the level of optic nerve head (Fig. 2m). In *Slc4a3* ^+/+^ mice, the optic nerve head consisted of well-aligned bundles (not shown).

**Figure 3:**
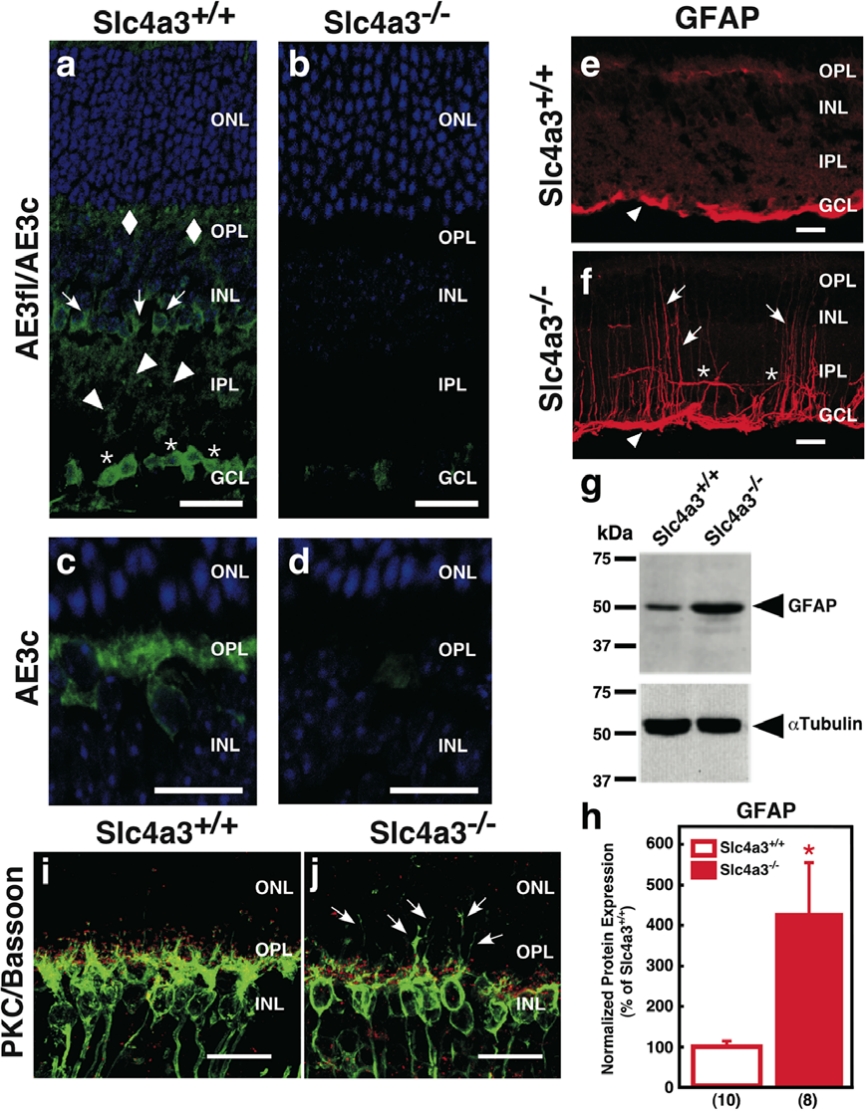
Localization of AE3fl and AE3c in mouse retina and retinal changes in *Slc4a3* ^−/−^ mice. (a–d) Retina cross sections of 4 months *Slc4a3* ^+/+^ mice (a, c), showing staining of the outer plexiform layer and Müller cells somas (arrows) and processes (arrowheads), and horizontal cells (diamond), using C-terminus antibody (a); somas in the ganglion cell layer were also immunolabeled. N-terminus antibody (specific to AE3c isoforms) labeled horizontal cell somas and dendrites (c). Specific staining was absent in 4 months *Slc4a3* ^−/−^ littermates (b, d) scale bar = 25 µm. GFAP was restricted to inner limiting membrane in *Slc4a3* ^+/+^ mice (e), while it stained radial (arrows) and tangential (stars) processes and was elevated in the inner limiting membrane (arrowheads, a and b) in age-matched *Slc4a3* ^−/−^ mice (f); scale bar = 20 µm. (g) Western blot analysis of protein samples (50 µg) prepared from whole retinal lysates of wild-type *Slc4a3* ^+/+^, and null *Slc4a3* ^−/−^ mice, resolved by 10% SDS-PAGE (*top panel*); α-tubulin served as a loading marker (*bottom panel*). (h) Summary of GFAP expression normalized to α-tubulin. Values are expressed relative to *Slc4a3* ^+/+^ protein levels. Brackets represent number of retinas analyzed. *Indicates statistically significant difference (*P*<0.05), compared to *Slc4a3* ^+/+^ wild type mice. Immunostaining of *Slc4a3* ^+/+^ (i) and null *Slc4a3* ^−/−^ mice (j) retinas examined by confocal microscopy using double-labeling for Bassoon (OPL, red) and PKC-α (rod bipolar cells, green). Arrows indicate sprouting of processes in the OPL of *Slc4a3* ^−/−^ mice; scale bar = 20 µm.

Confocal immunofluorescence (Fig. 3a–d) confirmed the expression of the two alternate AE3 forms [10], AE3 cardiac (AE3c), and AE3 full length (AE3fl), respectively in horizontal cells [somas in inner nuclear layer (INL) and processes in outer plexiform layer (OPL)], Müller cells (somas and processes in the inner nuclear layer, and processes in the inner plexiform layer), and somas in the ganglion cell layer of *Slc4a3* ^+/+^ but not *Slc4a3* ^−/−^ retinas. *Slc4a3* ^−/−^ mice over-expressed the intermediate filament protein GFAP in Müller cells and astrocytes (Fig. 3f; 6 months), but not in age-matched *Slc4a3* ^+/+^ mice (Fig. 3e). Elevation of GFAP levels in *Slc4a3* ^−/−^ retinas was confirmed on immunoblots (Fig. 3g, 3h; 4–8 months). Indeed, GFAP increased ∼3.5 fold in *Slc4a3* ^−/−^ mice, relative to wild type *Slc4a3* ^+/+^. Another pathologic hallmark was the aberrant sprouting of rod bipolar cell dendrites into the outer nuclear layer, evidencing that retinal remodeling does occur in *Slc4a3* ^−/−^ mice. Finally, the observation that the presynaptic marker bassoon undergoes down-regulation in AE3^−/−^ (Fig. 3i, 3j) indicates further pathological changes.

**Figure 4:**
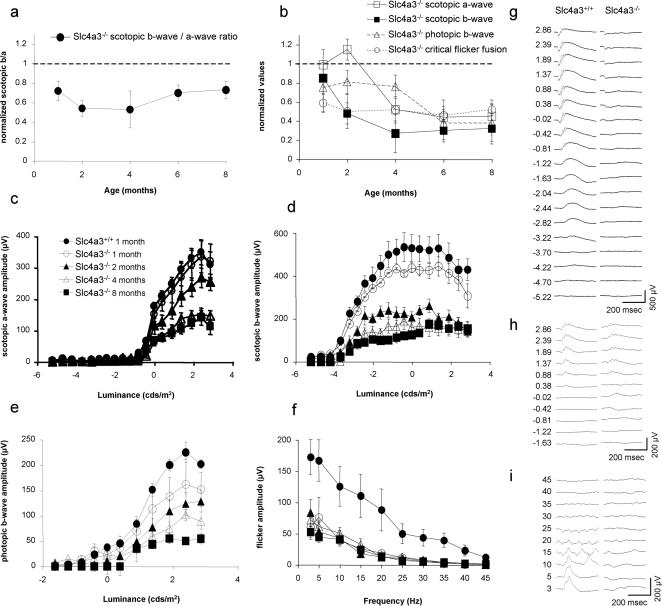
ERG responses in *Slc4a3* ^−/−^ mice. (a) Diminished ratios of ERG b-wave over a-wave maximal amplitudes for *Slc4a3* ^−/−^ mice under scotopic adaptation when normalized against age-matched *Slc4a3* ^−/−^ mice. Lowered b/a ratios indicate preferential b- over a-wave loss, and these differences were statistically significant at 1–8 months of age. (b) Decline with age of scotopic a-wave and b-wave amplitudes, as well as photopic b-wave amplitude and critical flicker fusion for *Slc4a3* ^−/−^ mice, normalized against age-matched *Slc4a3* ^−/−^ mice. In comparison with other parameters, scotopic a-wave amplitude began to decline at later ages. (c–e) Intensity response curves for scotopic (c) a-waves and (d) b-waves, as well as for (e) photopic b-waves. (f) Amplitude of flicker response in function of stimulus frequency under photopic adaptation. (g–i) Representative ERG traces obtained during intensity response series under (g) scotopic and (h) photopic adaptation, and during (i) flicker frequency series under photopic adaptation. For panels (a–f), results depict the mean ± s.e.m. For panels (g–i), units on the left are in (g–h) cds/m^2^, and (i) Hz.

To test for potential retina dysfunction in *Slc4a3* ^−/−^ mice, photoreceptor activity was distinguished from post-synaptic inner retina activity by measuring the electroretinogram (ERG) a-wave and b-wave, which respectively represent photoreceptor and inner retina activity. Reduction of any of these components implies visual losses. We found a pronounced and selective ERG b-wave depression up to ∼60%, that was age-independent (Fig. 4a, 4b, 4d, 4e, 4g, 4h). Another indicator of inner retina function, the flicker ERG, also paralleled b-wave reductions in *Slc4a3* ^−/−^ mice (Fig. 4f, 4i). Moreover, oscillatory potentials (wavelets on ascending b-wave), which also reflect inner retina functional integrity, were absent at all ages tested in *Slc4a3* ^−/−^ mice. In addition to specific inner retina defects at all ages in *Slc4a3* ^−/−^ mice, there was a progressive scotopic a-wave amplitude reductions (reaching statistical significance only by 4 months; see Fig. 4b, 4c, 4g) implying phototransduction failure in the already compromised vision of these mice, secondary to the ongoing inner retina defect.

**Figure 5:**
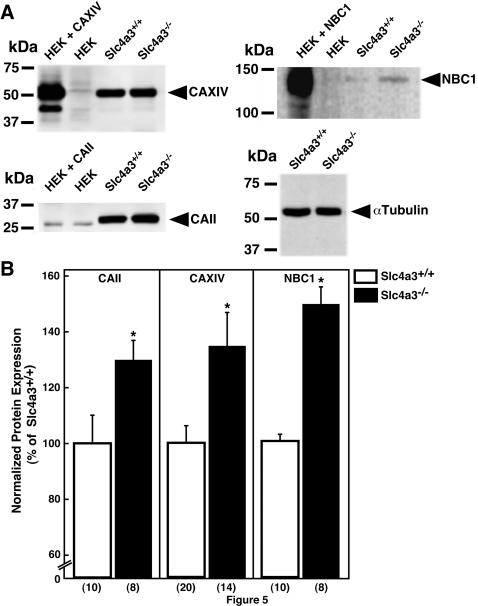
Expression of carbonic anhydrases II and XIV, and NBC1 Na^+^/HCO_3_^−^ cotransporter proteins in *Slc4a3* ^−/−^ mice. CAII, CAXIV, and NBC1 expression in adult *Slc4a3* ^−/−^ and wild type mouse retinal extracts, detected on immunoblots. (A) HEK293 cells individually transfected with CAII, CAXIV, and NBC1, cDNAs, and mock-transfected HEK293 cells, respectively, were used for positive and negative control of retinal immunoblots. Protein samples (50 µg) were probed with α-tubulin as a loading marker for *Slc4a3* ^−/−^ and *Slc4a3* ^+/+^ retinas. (B) Summary of the protein expression normalized to α-tubulin. Values are expressed relative to the *Slc4a3* ^+/+^ protein expression. The amount of CAII, CAXIV, and NBC1 protein is increased in the *Slc4a3* ^−/−^ mutant relative to wild type *Slc4a3* ^+/+^ mice. Brackets represent number of retinas analyzed. *Indicates statistically significant difference (*P*<0.05), compared to *Slc4a3* ^+/+^ wild type mice.

**Figure 6:**
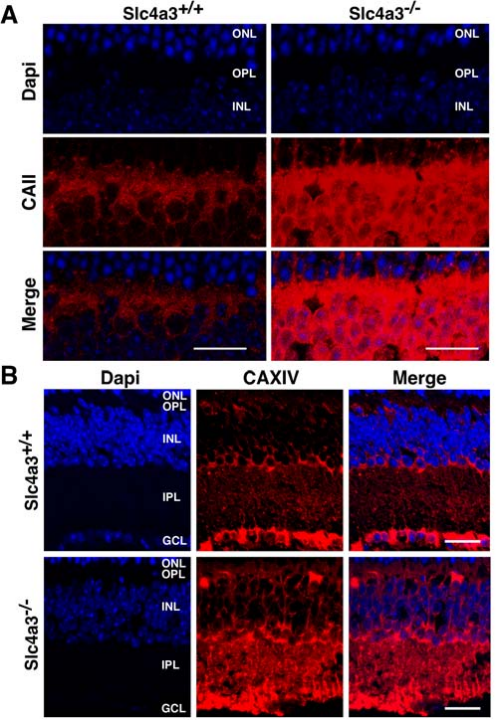
Localization of carbonic anhydrases II and XIV in *Slc4a3* ^−/−^ mouse retina. Frozen vertical sections of adult wild type *Slc4a3* ^+/+^ and *Slc4a3* ^−/−^ null mouse littermates retina were mounted in the same slide and labeled with goat anti-CAII ((A), 1∶100 dilution) or goat anti-CAXIV ((B), 1∶100 dilution) antibodies. Immunofluorescence signals were visualized by Alexa fluor 594-conjugated anti-goat IgG antibody (red, 1∶100 dilution). Sections were mounted in a DAPI media to identify nuclei, and images collected with a Zeiss LSM 510 laser-scanning confocal microscope with ×40/1.3 oil immersion objective (Neofluar oil). Merged images display CAII and CAXIV labeling overlapping nuclei staining. Scale bar = 20 µm. ONL = outer nuclear layer; OPL = outer plexiform layer; INL = inner nuclear layer; IPL = inner plexiform layer; GCL = ganglion cells layer.

AE3fl and AE3c proteins are found in a specific type of glial cells (Müller), and of neuronal cells (horizontal), respectively [10]. Both NBC1 [24] and carbonic anhydrase enzymes II (CAII) and XIV (CAXIV) [25], which are involved in bicarbonate metabolism and transport, are found in these same two cell types selectively expressing AE3. Therefore, we examined the expression of CAII (whole lysate), and CAXIV and NBC1 (membrane) cellular fractions of freshly isolated *Slc4a3* ^−/−^ null and *Slc4a3* ^+/+^ wild type mouse retinas (4–8 months), by immunoblotting. Samples were probed with α-tubulin as a loading marker. Cytoplasmic CAII, and CAXIV, with catalytic domain anchored to the extracellular surface, showed strong expression in *Slc4a3* ^−/−^ and *Slc4a3* ^+/+^ retinas (Fig. 5A). NBC1 was also detectable in Slc4a3^−/−^ and *Slc4a3* ^+/+^ retinas (Fig. 5A). Notably, expression of CAII, CAXIV, and NBC1 increased by 30%, 35%, and 50%, respectively, in *Slc4a3* ^−/−^ compared to *Slc4a3* ^+/+^ retinas (Fig. 5B). This abnormal elevation in CAII, CAXIV, and NBC1 (not shown) protein levels, was confirmed by immunohistochemistry combined with confocal microscopy, in retinas of *Slc4a3* ^−/−^ and *Slc4a3* ^+/+^ mouse (Fig. 6). Frozen vertical sections of *Slc4a3* ^−/−^ and *Slc4a3* ^+/+^ mouse retina were mounted on the same slide and used to study localization and immunoreactivity of CAII and CAXIV, in adult mice. CAII was found in horizontal cells, predominantly associated with cell membranes (somas in inner nuclear layer and processes in outer plexiform layer) (Fig. 6A), whereas CAXIV showed intense labeling of Müller cells (somas and processes in the INNER NUCLEAR LAYER, and processes in the inner plexiform layer), and somas in the ganglion cell layer (Fig. 6B), in both *Slc4a3* ^−/−^ and *Slc4a3* ^+/+^ mice. Interestingly, under same experimental and imaging capture conditions, immunoreactivity of CAII and CAXIV labeling in *Slc4a3* ^−/−^ null mouse was stronger than in wild type littermates, confirming elevated expression of CA proteins in the pathologic retinas.

**Figure 7:**
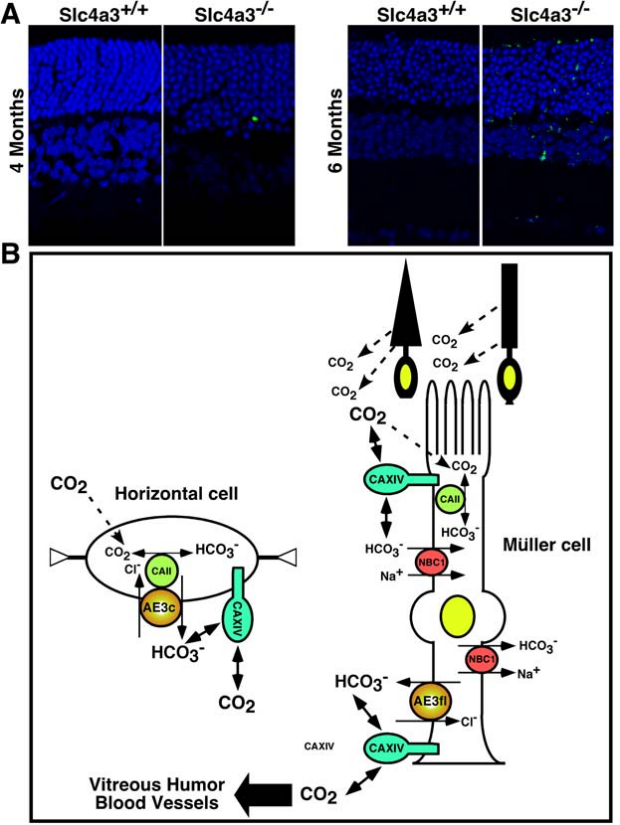
Apoptosis in retinas of *Slc4a3* ^−/−^ mice. (A) Absence of apoptotic nuclei in retinas of 4 and 6-month-old *Slc4a3* ^+/+^ mice. Nuclei (blue) are stained with DAPI dye. Apoptotic nuclei are shown (green) in retinas of age-matched *Slc4a3* ^−/−^ mice littermates, with predominant apoptotic nuclei found at 6-months of age. (B) Model demonstrating functional significance of AE3fl expression in Müller glial cells, and functional significance of AE3c in horizontal cells. In Müller cells, cytoplasmic carbonic anhydrase (CAII) can trap CO_2_ release by the photoreceptors intracellularly by converting to HCO_3_^−^ and H^+^. AE3fl located in Müller cells end feet facilitate the removal intro the vitreous or the blood of the HCO_3_^−^ and H^+^, in a process that is maximized by the CAXIV. In horizontal cells, AE3c may contribute to pH_i_ homeostasis by exchanging intracellularly produced HCO_3_^−^ with Cl^−^. CAII and CAXIV are also involved in tight regulation of horizontal cells pH_i_, increasing the effectiveness of the bicarbonate buffer system.

Increased expression of HCO_3_^−^ transporter (NBC1), and HCO_3_^−^ regulatory proteins (CAII, CAXIV) suggests the occurrence of compensatory changes in response to the loss of AE3 in *Slc4a3* ^−/−^ mouse retinas. Functional defects in the *Slc4a3* ^−/−^ null mice (ERGs) indicates that the ability to maintain acid–base balance is dramatically compromised in the inner retina and cannot be fully compensated by other pH regulatory proteins. We examined whether this leads to cell death in the retina. Staining for apoptotic cells, using TUNEL, revealed increased number of apoptotic nuclei from 4–6 months, in both the outer and inner nuclear retina layers, in *Slc4a3* ^−/−^ but not *Slc4a3* ^+/+^ retinas (Fig. 7A).

We have confirmed a key role played by the AE3 Cl^−^/HCO_3_^−^ exchangers in the mammalian retina, proving that bicarbonate transport and metabolism are key elements of normal ocular function. The specific visual phenotype resulting from AE3 deficiency, however, is unexpected, considering that the AE3 anion exchanger is expressed in other excitable tissues such as the heart and brain.

## Discussion

In this work, we have demonstrated that the apoptotic cascade is initiated as a result of absence of AE3-mediated HCO_3_^−^ fluxes. AE3 activity normally contributes to the regulation of pH_i_, [Cl^−^] and cell volume. In addition to contributing to cytoplasmic pH regulation in Müller glial cells, AE3 may act as an additional HCO_3_^−^ efflux mechanism working in concert with CAII and CAXIV to remove CO_2_ produced by photoreceptors. Along with AE3c, CAII and CAXIV are also involved in tight regulation of horizontal cells pH_i_, increasing the effectiveness of the bicarbonate buffer, forming metabolon-like systems [26] (Fig. 7B).

The consequence of AE3 deficiency is a unique visual defect phenotype associated with a deregulation in pH mechanisms: a selective inner retina defect followed at later ages by photoreceptor degeneration. As for the human counterpart, all aspects of retina dysfunction reported here share analogies with most forms of human vitreoretinal degeneration: selective reduction of scotopic ERG b-wave amplitude, optic nerve and retinal vessel anomalies, and sheathing of retinal vessels.

### Specific inner retina defect: pH implication

In the visual system, AE3 activity contributes to regulation of pH_i_, [Cl^−^], and cell volume. In addition to regulating pH_i_ in Müller glial and horizontal neuronal cells, AE3 may contribute to the removal of photoreceptor-generated CO_2_ waste.

Herein, we have demonstrated the occurrence of functional defects in the AE3^−/−^ null mice by electroretinogram (ERG) (Fig. 4). Potential retina dysfunction in AE3^−/−^ mice was evaluated by measuring ERG a-wave and b-wave, which respectively represent photoreceptor and inner retina functional activity. Reduction of any of these components implies visual losses. We found a pronounced and selective ERG b-wave depression that was present at all ages tested (1–8 months) in the AE3^−/−^ mice. Selective b-wave depression in AE3^−/−^ mice (evidenced by a drop in b/a ratio; Fig. 4a) is likely related to lower extracellular pH in the inner retina and reduced buffering power, following failed HCO_3_^−^ secretion by AE3. Consistent with this observation, ERG recordings from isolated toad retina indicate that acidification selectively suppresses the b-wave [27]. Small reductions in extracellular pH (on the order of 0.25) impede the function of the Cav1.4 presynaptic calcium channels [28], involved in synaptic transmission between photoreceptors and bipolar cells. Dysfunction in Cav1.4, such as occurs in congenital stationary night blindness (type 2), leads to selective b-wave diminution, with sprouting of bipolar cell dendrites [29] as reported here in *Slc4a3* ^−/−^ retina. Therefore, both the absence of synaptic inputs on aberrantly extended bipolar cell dendrites, and Cav1.4 dysfunction in the photoreceptor to bipolar cell synapses would contribute to selective b-wave reduction in *Slc4a3* ^−/−^ mice. Furthermore, loss of AE3 in Müller cells likely affects their function, impacting on b-wave generation such as seen in Müller cell sheen dystrophy [30]. Lack of AE3 in horizontal cells might also contribute to b-wave reductions, since exclusive loss of horizontal cells causes selective b-wave reductions [31]. In addition, horizontal cell inhibitory feedback to cones (responsible for receptive field antagonistic surround) is regulated by pH changes at the synaptic level. Changes in pH resulting from H^+^ accumulation modulate the presynaptic Cav1.4 voltage-gated calcium channels, in fish retina [32], and in transfected cells [28]. Also, reduction in flicker ERG and oscillatory potential amplitudes, at a time when scotopic a-waves remain unaffected, both support the occurrence of a selective inner retina defect. A progressive scotopic a-wave amplitude reduction, however, was observed in AE3^−/−^ null mice only at 4 month of age and later, suggesting initiation of photoreceptor dysfunction and potentially photoreceptor death (Fig. 4c). The delayed onset of photoreceptor pathologies could be attributable, in part, to prolonged Müller cell dysfunction [33]. The elevated expression of GFAP by Müller cells reflects the induction of gliotic responses characteristic of retinal degeneration. Müller cells are essential to retina ionic homeostasis and impairment of their supportive functions have been associated with retinal dystrophies [33].

### Ocular anomalies in AE3^−/−^ and CAXIV^−/−^ null mice

Functional abnormalities in AE3^−/−^ mice retinas were accompanied by increased expression of CAII and CAXIV enzymes, and NBC1 Na^+^/ HCO_3_^−^ co-transporter, suggesting the occurrence of compensatory changes for the loss of AE3 (Fig. 5, 6). While we found compensatory changes in the AE3^−/−^ knockout mice, the ability to maintain acid–base balance in the inner retina is likely to remain dramatically compromised given the phenotype of these mice.

Finally, the AE3^−/−^ null mice presented with late onset photoreceptor cell death (Fig. 7A). Presumably, following functional ERGs and anatomical abnormalities, the apoptotic cascade was initiated in the AE3^−/−^ null mice, as a result of absent AE3-mediated HCO_3_^−^ fluxes.

In the eye, CAXIV, a membrane bound isozyme, is expressed within the retina, in Müller glial cells and retinal epithelial cells [25], [34]. Because CAXIV is expressed in astrocytes, Müller cells, and the retinal epithelial cells, its expression spans the entire thickness of the retina. CAXIV distribution pattern is consistent with involvement in pH regulation and trans-retinal transport functions. Studies using CA inhibitors suggest that CAs, most likely CAII, CAIV and CAXIV, buffer the excess acidification in retina under certain metabolic conditions by preventing increase in H^+^ concentration [35]–[37]. Therefore, a role for CAXIV in buffering the subretinal space volume could be predicted.

A mouse deficient for CAXIV was recently described [37]. Of interest, flash ERGs performed at 2, 7, and 10 months of age showed that the rod/cone a-wave, b-wave, and cone b-wave were significantly reduced (up to 45%) in the CAXIV^−/−^ compared to wild-type mice [38], matching with findings of AE3^−/−^ mice. Moreover, reductions in the dark-adapted response were not progressive between 2 and 10 months, as reported here for AE3^−/−^ mice, suggesting a functional linked between AE3 and CAXIV in the normal eye function. Although CAXIV^−/−^ and AE3^−/−^ null mice shared very similar abnormal electrophysiological responses in the eye, the more profound pathology observed in the AE3^−/−^ mice suggests additional roles played by the AE3 Cl^−^/HCO_3_^−^ exchanger in the retina.

Finally, we have found that the AE3/CAXIV forms a physical and functional complex in the mouse retina (Alvarez et al. Manuscript in preparation). The AE3-mediated HCO_3_^−^ flux is maximized by CAXIV interaction. Tethering of CAXIV [39] and CAII [12] to AE3 maximizes the transmembrane HCO_3_^−^ gradient local to AE3, thereby activating transport rate. Association of the AE3 Cl^−^/HCO_3_^−^ exchanger and carbonic anhydrases II and XIV may represent a mechanism for the disposal of high CO_2_ and H^+^ production, and for pH regulation in the inner retina and brain. Recurrent phenotypes found for AE3^−/−^ and CAXIV^−/−^ null mice, and on direct interaction of AE3 and CAXIV, suggest that the association of AE3 Cl^−^/HCO_3_^−^ exchanger and CAXIV enzyme play a key role in normal ocular function.

### Clinical implications

To date, only one human variant (Ala867Asp substitution), has been identified in the *SLC4A3 gene* [21], [40]. The Ala867Asp variant was associated with common subtype idiopathic generalized epilepsy (IGE) in humans [40]. The effect of the variant on AE3 function has not been assessed. Eye examination and heart function of patients with the *SLC4A3* Ala867Asp variant resulting in IGE have yet to be reported.

This paper provides the first documentation for a role of AE3 in normal ocular function. In addition, our findings demonstrate that lack of AE3 causes retinal pathology stereotypical of most vitreoretinal degenerations. Therefore *SLC4A3* (AE3) is a novel candidate gene that must now be considered for the diagnosis of HVD.

HVD are a complex group of genetic disorders manifesting abnormalities of the vitreous and retina, affecting approximately 1 in 3000 people [41]. HVD includes lattice degeneration, snowflake vitreoretinal degeneration, X-linked juvenile retinoschisis, vitreoretinopathies (chromosome 5q, e.g. Wagner syndrome), chondrodysplasias (e.g. Stickler syndrome), and enhanced S-cone syndrome/Goldmann-Favre vitreotapetoretinal degeneration [42]–[48]. HVD can be difficult to distinguish because of subtle and overlapping clinical findings. Nevertheless, nearly all HVD present with altered ERG, in particular a reduction or absence of the scotopic ERG b-wave, different degrees of retinal lesions, poor visual acuity, and retinal blood vessel abnormalities [49]. Abnormal development of the vitreous progresses to increased vitreoretinal traction and retinal detachment, with a concomitant loss of vision. Additional ocular and systemic features of HVD may occur, depending on the underlying cause.

Our results provide compelling evidence linking AE3 Cl^−^/HCO_3_^−^ exchangers, pH imbalance in the inner retina, and vitreoretinal degeneration.
